# A postal survey of maternal sleep in late pregnancy

**DOI:** 10.1186/1471-2393-12-144

**Published:** 2012-12-10

**Authors:** B Lynne Hutchison, Peter R Stone, Lesley ME McCowan, Alistair W Stewart, John MD Thompson, Edwin A Mitchell

**Affiliations:** 1 Department of Paediatrics, The University of Auckland, Auckland, New Zealand; 2 Department of Obstetrics and Gynaecology, The University of Auckland, Auckland, New Zealand; 3 School of Population Health, The University of Auckland, Auckland, New Zealand

**Keywords:** Pregnancy, Sleep disturbances, Sleep quality, Snoring, Daytime sleepiness

## Abstract

**Background:**

Sleep disturbances in late pregnancy are common. This study aimed to survey sleep problems in third trimester pregnant women and to compare sleep in the pre-pregnancy period with the third trimester.

**Methods:**

Third-trimester women (n=650) were sent a postal survey containing questions relating to sleep experience, including perceived sleep quality, sleep difficulties, night waking, sleep environment, snoring, daytime tiredness and daytime napping. Time periods reported on were before pregnancy and in the last week.

**Results:**

Respondents numbered 244 (38%). Before pregnancy, the mean reported duration of night-time sleep was 8.1 (SD 1.1) hours; in the last week this had decreased to 7.5 (SD 1.8) hours (p<.0001). Only 29% rated their sleep quality in the last week as very good or fairly good, compared with 82% rating their sleep this way before the pregnancy. The main reasons for sleeping difficulties were discomfort (67%) and pain (36%). Snoring increased significantly over the course of the pregnancy, with 37% reporting snoring often or every night in the last week. Those with a pre-pregnancy body mass index of greater than 25 were significantly more likely to snore (p=.01). Only 4% of women had an abnormal Epworth Sleepiness Scale score (i.e. >10) prior to pregnancy, whereas in the last week 33% scored in the abnormal range. Likewise, 5% had regularly napped during the daytime before pregnancy, compared with 41% in the last week.

**Conclusions:**

Sleep problems are common in women in late pregnancy, and increase markedly compared with before pregnancy.

## Background

Sleep problems in pregnancy are common, with most pregnant women reporting declining sleep quality and increased night waking especially in the third trimester [1-6]. Studies have found that 82-98% of women in late pregnancy report waking at night [2,3,6], and 64% - 86% report problems with sleep quality during pregnancy [4]. Insomnia and increased wakefulness after sleep onset can result from a large number of potential causes including gastro-oesophageal reflux, discomfort, frequent micturition, and dyspnoea [1,2,5]. Poor sleep quality and lowered sleep duration have been associated with a higher incidence of preterm birth [7] and glucose intolerance and gestational diabetes in pregnancy [8,9]. Furthermore, a number of studies have shown an increased frequency of snoring and sleep disordered breathing as pregnancy progresses which may contribute to the increased incidence of glucose intolerance [9] and pregnancy induced hypertension [10].

This study aimed to assess sleep practices and sleep quality in women in the last trimester of pregnancy, and to document self-reported changes that occurred between the pre-pregnancy period and the last trimester of pregnancy.

## Methods

In September 2011, survey questionnaires were posted to 650 pregnant women randomly selected from all women (n=1597) who were between 27 and 38 weeks gestation on the booking list at National Women’s Health (NWH), a large maternity unit in Auckland, New Zealand. The questionnaire contained questions relating to their sleep experience both before becoming pregnant and in the last week. Questions included maternal demographics, perceived sleep quality, sleep difficulties, night waking, snoring, daytime tiredness, and daytime napping. Questions relating to sleep quality were based on the Pittsburgh Sleep Quality Index (PSQI), a 9-question self-rated test intended to measure sleep disturbances and quality of sleep [11]. Sleep disordered breathing questions were based on the Berlin Sleep Questionnaire (BSQ), a 10-item screening test designed to predict sleep apnea and sleep disordered breathing [12]. Participants also completed the Epworth Sleepiness Scale (ESS) to assess daytime sleepiness [13]. The ESS asks people to rate, on a 4-point scale (0 – 3), their usual chances of dozing off or falling asleep in eight different situations or activities that most people engage in as part of their daily lives. The total ESS score is the sum of 8 item-scores and can range between 0 and 24. The higher the score, the higher the person’s level of daytime sleepiness. We did not use questions from the PSQI and BSQ relating to blood pressure and areas already covered in the ESS. Additional questions were also posed in order to expand understanding of the respondents’ experience specific to pregnancy. Additionally, the questionnaire asked the mother to report on her height, pre-pregnancy weight, ethnicity, smoking status at the time of the survey, parity and due date.

Statistical analysis was carried out using SAS (Version 9.1, SAS Institute Inc., Cary, NC, USA). Chi-squared analysis was used for categorical variables and t-tests were performed for continuous variables. The study complied with the Helsinki Declaration and was approved by the NZ Ministry of Health’s Northern Regional X Ethics Committee; reference number NTX/11/EXP/172.

## Results

In total, 650 survey forms were mailed; 252 were returned. Eight forms were excluded as these women reported they had already delivered their infants by the time they received the questionnaire, leaving 244 (38%) completed surveys included in the study. Apart from gestational age, no information was available on the non-respondents. Respondents were mostly of NZ European ethnicity, with a median age of 33 years and a median gestation of 36 weeks. Forty-five percent had no previous children; a further 37% had one child and 15% had two children. Characteristics of the respondents are outlined in Table 1.

**Table 1:** Characteristics of respondents

***Characteristic***	***Category***	***n (%) or mean (SD)***
Maternal age (missing=3)	<20	6 (2.5)
	20-34	138 (57.3)
	>35	97 (40.3)
Ethnicity (missing=3)	European	150 (62.2)
	Other	59 (24.5)
	Maori	17 (7.1)
	Pacific	15 (6.2)
Marital status (missing=40)	Married	168 (82.3)
	De facto	28 (13.7)
	Single	8 (3.9)
Current smoker (missing=4)	Yes	10 (4.2)
Sleep with partner (missing=0)	Yes	200 (82.0)
Gestation (weeks) (missing=11)		35.0 (2.7)
Gestation, categorised (weeks)	28 to<32	25 (10.8)
	32 to <34	49 (21.1)
	34 to <36	42 (18.1)
	36 to <38	73 (31.5)
	≥38	43 (18.5)
Parity (missing=6)	0	108 (45.4)
	1	87 (36.6)
	2	35 (14.7)
	≥3	8 (3.4)
Pre-pregnancy BMI, calculated (missing=19)		23.8 (4.5)
Pre-pregnancy BMI categorised (missing=19)	<18.5	6 (2.7)
	18.5 – 24.9	159 (70.7)
	25.0 – 29.9	36 (16.0)
	≥30	24 (10.7)

### Sleep duration and quality

Before pregnancy, the mean number of hours of night-time sleep was reported by mothers to be 8.1 (SD 1.1) hours; in the last week this had decreased to 7.5 (SD 1.8) hours (p<.0001). Thirty-two percent reported sleeping less than 7 hours per night and 16% had slept less than 6 hours per night in the last week, compared with reported rates of 9% and 2% respectively before pregnancy (p=<.0001). Using paired t-tests, it was seen that overall the participants’ mean sleep duration had reduced by a mean of 35.8 (SE 0.10) per night minutes in the last week compared with before pregnancy (p=.0001). Individually however, 31% of women reported an increase in night time sleep hours.

Perceived sleep quality deteriorated over the course of the pregnancy, with 82% rating their sleep quality as very good or fairly good before pregnancy, compared with 29% rating their sleep this way in the last week. Thirty-eight percent rated their sleep as fairly bad or very bad in the last week, compared with only 4% before pregnancy. Likewise, in the last week 96% of respondents reported needing to go to the toilet during the night and for 65% of these women this was at least twice a night. Difficulty falling asleep and getting back to sleep, restless sleep, and needing to change sleep positions during the night were all problems that worsened during the pregnancy (Table 2).

**Table 2:** Sleep quality

***Variable***	***Response***	***Before pregnancy n (%)***	***In last week n (%)***	***P-value***
Difficulty falling asleep	Never/rarely/			
	sometimes	218 (90.1)	180 (74.4)	<.0001
	Often/every night	24 (9.9)	62 (25.6)	
Difficulty getting back to sleep	Never/rarely/			
	sometimes	224 (92.4)	145 (59.7)	<.0001
	Often/every night	19 (7.8)	98 (40.2)	
Wake up at night	Yes	66 (27.2)	234 (96.3)	<.0001
Go to toilet at night	Yes	101 (41.9)	232 (95.8)	<.0001
If yes, number of times up to toilet	1	82 (86.3)	77 (35.0)	<.0001
	2	7 (7.4)	66 (30.0)	
	≥3	6 (6.4)	77 (35.1)	
Are you a restless sleeper?	No/a little/average	211 (87.2)	105 (43.3)	<.0001
	More than average	19 (7.8)	76 (31.4)	
	Very restless	12 (5.0)	61 (25.2)	
Changed sleep position at night	Not lots*	196 (80.9)	105 (43.8)	<.0001
	Lots	46 (19.0)	135 (56.3)	
Sleep quality self-rating	Very good	124 (51.2)	17 (7.0)	<.0001
	Fairly good	75 (31.0)	52 (21.5)	
	Average	33 (13.6)	81 (33.5)	
	Fairly bad	10 (4.1)	74 (30.6)	
	Very bad	0	18 (7.4)	

* This category consists of the following subcategories: 0, possibly once, possibly twice, >twice but not lots.

**Figure 1:**
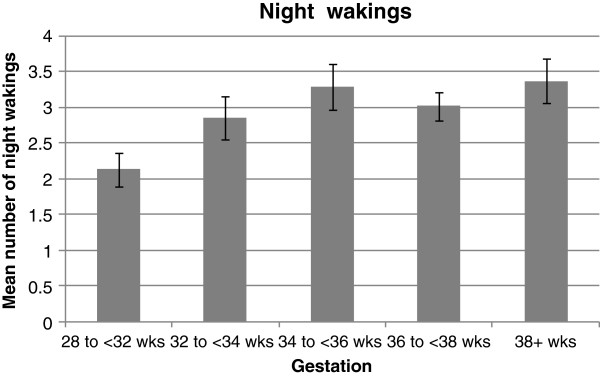
**Mean number of times waking at night at each gestational age.** Error bars denote standard error of mean.

Women in the later stages of the last trimester woke significantly more often at night than those in the earlier stages (p=0.04). Compared to those at 28 to 32 weeks gestation (mean wakings 2.1 [SD 1.2]), the number of reported night wakings of women at 32 to 36, 34 to 36, 36 to 38 and ≥38 weeks was significantly higher, at 2.8 (SD 1.5), 3.3 (SD 2.0), 3.0 (SD 1.6), and 3.4 (SD 2.1) respectively (Figure 1).

### Reasons for difficulty sleeping

Before pregnancy, the predominant reason for having trouble sleeping was “things on my mind” (51%); however, by the third trimester, this was superseded by discomfort (67%), although pain and “things on my mind” still featured in 36% of respondents. Other physical discomforts such as heartburn, feeling hot, and trouble breathing comfortably were also significantly increased in the last week (Table 3). When asked “What usually wakes you in the morning?” the most common reason in the last week was the need to go to the toilet (33%), whereas before pregnancy this accounted for only 3% of awakenings.

**Table 3:** Reasons for difficulty sleeping

***Reason***	***Before pregnancy n (%)***	***In the last week n (%)***	***P-value***
Uncomfortable	13 (5.4)	163 (67.1)	<.0001
Pain	5 (2.1)	87 (35.8)	<.0001
Things on my mind	123 (50.6)	87 (35.8)	0.001
Heartburn	1 (0.4)	69 (28.4)	<.0001
Too hot	28 (11.5)	70 (28.8)	<.0001
Can’t breathe comfortably	4 (1.6)	56 (23.0)	<.0001
Partner snoring	69 (28.4)	46 (18.9)	0.01
Noise	34 (14.0)	19 (7.8)	0.03
Too cold	29 (11.9)	14 (5.8)	0.02
Partner turning	25 (10.3)	16 (6.6)	0.14
Bad dreams	32 (13.2)	37 (15.2)	0.51
Cough	21 (8.6)	27 (11.1)	0.36
Other	13 (5.4)	30 (12.4)	0.007

### Snoring

One hundred and fifty three women (63%) had ever been told they snored. Snoring increased significantly over the course of the pregnancy, with 28% reporting snoring three or more nights in the last week compared with 4% prior to pregnancy (Table 4). Snoring loudness also increased over this period, but being told that they had stopped breathing while snoring was reported in very few women either before pregnancy or in the last week. Of those who snored in the last week, 58% said it did not bother others. Those with a pre-pregnancy body mass index (BMI) ≥25 were significantly more likely to snore than those whose BMI was <25 (78% vs 59% respectively; p=.01).

**Table 4:** Snoring before pregnancy and in the last week

***Question***	***Response***	***Before pregnancy n (%)***	***In last week n (%)***	***P-value***
How frequently do you snore?	No snoring	116 (51.6)	103 (47.7)	<.0001
	1 – 2 nights per week	99 (44.0)	52 (24.1)	
	3 or more nights per week	10 (4.4)	61 (28.2)	
	Missing	19	28	
How loud is your snoring?	No snoring	119 (55.1)	103 (49.5)	<.0001
	Minimal / soft	77 (35.6)	47 (22.6)	
	As loud as talking / very loud	20 (9.3)	58 (27.9)	
	Missing	28	36	

### Daytime sleepiness

Approximately half of the mothers were still tired after their night sleep compared with only 10% of them reporting that they had felt this way before pregnancy. In the last week, 41% had napped during the daytime, with the median nap time being 90 minutes [IQR=(60, 120)]. However, the total reported hours of sleep per 24 hours (daytime naps plus night-time sleep hours) before pregnancy and in the last week did not differ significantly: 8.6 (SD 1.3) versus 8.7 (SD 2.0) hours respectively.

Paired t-tests of the total scores on the Epworth Sleepiness Scale revealed that women’s Epworth scores reduced overall by 4.4 (SE 0.28, p<0.0001) in the period between prior to pregnancy and the last week. Only 6.5% of women had an increased Epworth score in the last week compared with before pregnancy. When categorised according to the official ESS criteria (i.e. total score of 0 – 10 is normal and greater than 10 is abnormal)[14], 4% of women had an abnormal score prior to pregnancy, whereas 33% scored in the abnormal range in the last week (Table 5).

**Table 5:** Daytime sleepiness, daytime napping and ESS score

***Question***	***Response***	***Before pregnancy n (%)***	***In last week n (%)***	***P-value***
How often are you tired after night sleep? (missing=6)	0 – 2 times a week	213 (89.5)	113 (47.1)	<.0001
	3 – 7 times a week	25 (10.5)	127 (53.0)	
How often are you tired during day? (missing=6)	0 – 2 times a week	218 (91.6)	95 (40.1)	<.0001
	3 – 7 times a week	20 (8.4)	142 (59.9)	
How often do you nap during day? (missing=4)	0 – 2 times a week	229 (95.4)	143 (59.3)	<.0001
	3 – 7 times a week	11 (4.6)	98 (40.7)	
Categorised ESS total score	Normal (0 – 10)	222 (96.1)	157 (67.1)	<.0001
	Abnormal (>10)	9 (3.9)	77 (32.9)	

ESS = Epworth Sleepiness Scale.

## Discussion

Sleep problems in late pregnancy are common. In this study we have provided an in-depth survey of sleep practices and symptoms in the third trimester of pregnancy and compared this with pre-pregnancy sleep in the same women.

Compared with pre-pregnancy, and consistent with other studies [2,3,6,9,15], we have shown a reduction in maternal sleep quality in the third trimester compared with before pregnancy. Much of the reduction in quality appears to be the result of night waking, which was almost universal in this group of mothers, particularly in the latter part of the third trimester, for reasons of nocturia, discomfort, pain and general restlessness.

Sleep duration decreased in the third trimester compared with before pregnancy, dropping from a mean of 8.1 hours to 7.5 hours a night. This contrasts with Hedman et al. [6] whose Finnish respondents reported a sleep duration of 7.8 hours both before pregnancy and in the third trimester. Facco et al. in Chicago analysed self-administered questionnaires from women in early pregnancy and again in the third trimester and showed that mean sleep duration dropped from 7.4 hours early in pregnancy to 7 hours in late pregnancy [9]. In a small Taiwanese study using actigraphy to measure sleep duration in nulliparous women the mean duration of total night time sleep in the third trimester was 6.4 (± 1.0) hours [16].

Those who reported snoring often or every night in the last week comprised 28%, slightly higher than others have shown using self-reported data (10% - 25%) [1,2,6]; however, Izci et al. used partner reports to determine the presence and frequency of snoring, and found that 35% to 39% were frequent snorers [17,18]. That group also investigated the pathophysiology of snoring and showed that in the third trimester of pregnancy the upper airways are significantly narrower than in non-pregnant women [18], and that the difference was only in the seated position but not when the subjects were supine [19]. This possibly acts as a protective mechanism against the effects of supine position on upper airways in late pregnancy.

Daytime fatigue also increased significantly over the course of the pregnancy with 33% of third-trimester women having an ESS total score >10. Consistent with this finding, Pien et al. from the University of Pennsylvania showed that in the month of delivery 45% of pregnant women scored >10 [4] while in a Scottish study 23% of third trimester women scored >10 [17]. In an Australian study, 11% of non-pregnant adults without evidence of a chronic sleep disorder scored >10 [14]. Whether variations in sleep duration and daytime fatigue relate to local lifestyle factors in different parts of the world is unknown.

Apart from the mechanical and physical effects of increased size and weight in late pregnancy, especially as these affect general fatigue and sleep disordered breathing, sleep in pregnancy may also be influenced by hormonal changes. While these changes were not measured in this survey, they are undoubtedly important. The hormones oestrogen, which decreases REM sleep, and progesterone, which increases non-REM sleep, ventilation and respiratory alkalosis, both increase markedly in pregnancy. In addition, a lowered cortisol-melatonin ratio in late pregnancy can result in poor quality of sleep [20]. It is important to consider however, that at this time in a woman’s life it may not only be hormonal or physiological factors that are at play. For instance, although sleep duration decreased overall, 31% of the women reported increased sleep duration, perhaps due to stopping work, changing childcare demands, other lifestyle factors, or the perceived need to sleep more because of increased fatigue.

While pregnant women and their obstetric carers may regard pregnancy sleep disorders as normal and to be endured, there is evidence that disrupted sleep and changing sleep practices may influence adverse pregnancy outcomes, and several reviews have commented on this [5,7,21]. An increased risk of preterm delivery [7,22] and postpartum depression [7] have been linked with sleep deprivation, while sleep disordered breathing is associated with gestational diabetes [8,9,21], low birth weight, preterm and small for gestational age infants, caesarean section and preeclampsia [23]. In addition, short sleep duration and severely disrupted sleep are also more likely to be associated with unplanned caesarean deliveries after controlling for infant birth weight [24]. Increased daytime napping and fewer toilet visits at night have been implicated as risk factors for stillbirth [25]. Whilst Stacey et al. reported that daytime sleepiness per se was not related to the incidence of stillbirth [25], increased daytime napping has been associated with sleep disordered breathing, at least in the non-pregnant population [26], and it is not uncommon for patients to underestimate their levels of daytime sleepiness. The current study does not include polysomnography or measures of fetal outcomes, so we are unable to comment on any potential associations between excessive daytime sleepiness, sleep disordered breathing and fetal health. Interventions to improve sleep and possible adverse pregnancy outcomes may thus be important and further studies are warranted to investigate this area.

The main limitation of this study was the retrospective nature of the questions leading to the possibility of recall bias, particularly for the questions relating to sleep prior to pregnancy. Preferably, the same survey administered to the women both before pregnancy and in the third trimester might have provided a more accurate picture of increased prevalence of sleep related behaviours of pregnancy. However, this would have increased the complexity of the study. Ideally, a polysomnogram would confirm the accuracy of maternal report.

In addition, there is no validated questionnaire for sleep disorders in pregnancy, and although we used questions from other validated tools such as the Pittsburgh Sleep Quality Index, the Berlin Sleep Questionnaire, and the Epworth Sleepiness Scale questionnaires, the relative contributions of common symptoms of pregnancy and true sleep disordered breathing are unclear. In fact, the Berlin questionnaire has been shown to poorly predict sleep disordered breathing in pregnant women [27]. Tiredness and reduced daytime functioning are frequently experienced by pregnant women [18,28], and this potentially compounds the problem of assessing the true prevalence of sleep problems in pregnancy. The validity of retrospective reporting of sleep questions prior to pregnancy has also not been established, although one study in a non-pregnant population has shown a high correlation between baseline ESS and a retrospective re-test 5 months later [29].

Another limitation may be that only 27% of our respondents were in the overweight or obese BMI categories, compared with 48% who were overweight or obese in the 2010 NWH annual report [30]; therefore, the incidence of sleep disordered breathing in our study may be lower than in the NWH population. Thus our results for snoring are likely conservative. Unfortunately we did not have access to information regarding gestational hypertension, pre-eclampsia, or gestational diabetes in the participants and so cannot comment on these conditions in relation to the sleep findings. Notwithstanding these limitations, this study has provided a clearer understanding of the problems, difficulties, and sleep practices experienced by third trimester pregnant women.

## Conclusions

Sleep problems were common in this group of women in late pregnancy, and these disturbances were increased comparative to their pre-pregnancy experience. Evidence is emerging that sleep quality, sleep disordered breathing and sleep practices can affect maternal-fetal outcomes and it appears that improving these areas might lessen the impact of adverse fetal outcomes. However, definitive standards of good sleep hygiene for women experiencing the physiological and hormonal changes of late pregnancy have not yet been determined and should be the subject of future research.

## Abbreviations

BMI: Body mass index; NWH: National Women’s Health; ESS: Epworth Sleepiness Scale; SD: Standard deviation; SE: Standard error; IQR: Inter-quartile range.

## Competing interests

The authors declare that they have no competing interests.

## Authors’ contributions

BLH developed the questionnaire, carried out the data collection and data entry, performed basic statistical analysis, and drafted the manuscript. LMEM and PRS participated in the planning of the study and contributed to the manuscript. AWS and JMDT contributed to the statistical analysis. EAM conceived of the study, participated in its design and oversaw the project. All authors read and approved the final manuscript.

## Pre-publication history

The pre-publication history for this paper can be accessed here:

http://www.biomedcentral.com/1471-2393/12/144/prepub
